# The risk association between systemic lupus erythematosus and glaucoma: a systematic review and meta-analysis

**DOI:** 10.3389/fimmu.2026.1808105

**Published:** 2026-06-22

**Authors:** Xue Li, Fang Wang, Liming Zhu, Xi Tian, Xi Yang

**Affiliations:** Department of Ultrasound Medicine, The Shapingba Hospital, Chongqing University (People’s Hospital of Shapingba District, Chongqing), Chongqing, China

**Keywords:** glaucoma, meta-analysis, risk, systematic review, systemic lupus erythematosus

## Abstract

**Background:**

Systemic Lupus Erythematosus (SLE) is a systemic autoimmune disease that may damage multiple organs. About one-third of SLE patients have ocular manifestations. SLE as a potential risk factor for glaucoma has attracted attention, but existing study conclusions remain controversial. This meta-analysis aimed to evaluate the risk association between SLE and glaucoma and provide supportive evidence for the comorbidity follow-up of SLE patients.

**Methods:**

The PubMed, EMBASE and Web of Science databases from the establishment of the database to November 2025 were retrieved, and studies measuring the association between SLE and glaucoma risk by hazard ratio (HR), odds ratio (OR), relative risk (RR), incidence rate ratio (IRR) and 95% confidence interval (CI) were included. Two researchers independently screened the literature, extracted the data and evaluated the risk of bias in the included studies. Meta-analysis was conducted using RevMan 5.3 software.

**Results:**

Five cohort studies were included, involving 5516709 participants. Meta-analysis showed that SLE was associated with an increased risk of glaucoma (OR = 2.85, 95% CI: 1.26–6.47, *P* = 0.01). Subgroup analyses showed stronger associations in studies with fewer covariate adjustments and in those that did not adjust for corticosteroid use.

**Conclusions:**

Existing evidence suggests that SLE may be associated with an increased risk of glaucoma. Given the limitations of this study, more prospective studies with rigorous design and full control of confounding factors are needed for further verification.

**Systematic review registration:**

https://www.crd.york.ac.uk/prospero, identifier CRD420261303001.

## 1 Introduction

Systemic Lupus Erythematosus (SLE) is a chronic autoimmune disease (1). It is mainly prevalent among women of childbearing age, and the age of onset ranges from childhood to over 70 years (1). Its characteristics are multi-system involvement and diverse clinical manifestations (1).Epidemiological data showed that the annual incidence rate of SLE varied regionally worldwide, with a reference range of approximately 1.4 to 11.0 cases per 100,000 people (2). Previous evidence indicated that ocular involvement in SLE was quite common among patients, with a prevalence rate that reached approximately one-third (3). This disease can directly damage multiple anatomical structures of the eye through immune-mediated mechanisms, involving the retina (such as vasculitis), lacrimal glands, choroid, optic nerve (such as optic neuritis), as well as anterior structures such as the episclera and sclera (4, 5). In addition to the ocular changes caused by SLE, the drugs used for systemic treatment of this disease also have potential hazards to the eyes (6).

Glaucoma, a disease characterized by progressive damage to the optic nerve, is the leading cause of irreversible vision loss worldwide (7). Its early clinical manifestations are often atypical, but it can lead to irreversible damage to the structure and function of the optic nerve, thus constituting an important potential cause of blindness in the SLE patient population (8). In addition to the retinal vasculitis and optic neuritis that may be caused by the disease itself in SLE patients, a more common and hidden threat comes from the long-term maintenance treatment drugs - glucocorticoids (9, 10). Some studies have found that the incidence of secondary glaucoma in SLE patients who have used glucocorticoids for a long time is significantly higher than that in the general population (6, 9, 10).

Currently, there is controversy regarding the association between SLE and the risk of glaucoma. Some cohort studies suggested that the risk of glaucoma was significantly increased in SLE patients. However, other studies have not found a statistically significant association. This study quantitatively assessed the strength of the association between SLE and the risk of glaucoma onset through systematic reviews and meta-analyses, thereby providing evidence base for the screening of complications and long-term management of SLE patients.

## 2 Methods

This systematic review was conducted in accordance with the Preferred Reporting Items for Systematic Reviews and Meta-Analyses (PRISMA) 2020 statement (11, 12). This meta-analysis was registered on the PROSPERO platform (CRD420261303001).

### 2.1 Search strategy

This study systematically retrieved the literature on the association between SLE and the risk of glaucoma from database inception (PubMed, Embase, and Web of Science) to November 2025. These studies reported the hazard ratio (HR), odds ratio (OR), relative risk (RR), or incidence rate ratio (IRR) along with their 95% confidence intervals (CI). The search strategy combined MeSH terms and free words. The search strategy was constructed by combining the relevant terms “Lupus Erythematosus, Systemic” and “Glaucoma” with Boolean logical operators “AND” and “OR”. In addition to the database search, the references of the included studies were manually traced to supplement the acquisition of potential related studies. The detailed search strategy can be found in the supplementary material (**Supplementary Table 1**).

### 2.2 Inclusion and exclusion criteria

#### 2.2.1 Inclusion criteria

This study included observational studies that met the following criteria:

1. Participants: The exposed group (or case group) consists of patients diagnosed with SLE, while the control group comprises individuals without SLE;
2. Study Design: Cohort study or case-control study;
3. Outcomes: A study on the association between SLE and the risk of new-onset glaucoma events. The glaucoma events must occur naturally during the observation period.
4. Data Requirements: It was necessary to provide the effect size that measures the strength of the association and its 95% confidence interval. Acceptable effect sizes included HR, OR, RR or IRR.

#### 2.2.2 Exclusion criteria

1. The same data only retains the version with the most complete information;
2. Case reports, reviews, comments, conference summaries and basic research;
3. The full text could not be obtained, or the key statistical information was missing or could not be converted.

### 2.3 Data extraction

All the retrieved literature was imported into the EndNote literature management software for organization. Firstly, duplicate records were removed through the built-in functions and manual verification of the software. The literature screening followed the following process: Two researchers independently conducted the initial screening of the included literature. Studies that clearly did not meet the inclusion criteria were excluded based on the title and abstract. Then, the full texts of the initially screened studies were obtained, and both researchers independently reviewed the full texts to determine whether they should be included. In case of disagreement during the screening process, a third senior researcher adjudicated. Data from the final included studies were independently extracted by the two researchers. The extracted contents included: the first author, publication year, start and end time of the study, country, research design, sample size, age, SLE diagnostic criteria and glaucoma diagnostic criteria, adjusted confounding factors, reported effect size and its 95% confidence interval.

### 2.4 Biased risk assessment

The study used the Risk of Bias in Non-Randomized Studies of Exposures (ROBINS-E) to assess the methodological quality of the included literature. The assessment process was independently carried out by two researchers, and judgments were made in the following seven core bias domains: confounding bias, selection bias of study subjects, bias in exposure classification, bias in exposure implementation, loss to follow-up and data missing bias, outcome measurement bias, and selective reporting bias. Each domain was rated as low, moderate, or high risk of bias according to the ROBINS-E guidelines. If there was a disagreement between the two researchers’ evaluations, a third senior researcher would intervene to arbitrate until a consensus was reached.

### 2.5 Data synthesis and analysis

A Meta-analysis was conducted using RevMan 5.3 software. Given the relatively low incidence of glaucoma, the various effect sizes (OR, HR, RR, IRR) included in the research reports were highly similar in numerical terms, all reflecting similar relative risks (13–16). All effect estimates were log-transformed to ensure normality and statistical comparability, and analyses were pooled using the DerSimonian-Laird random-effects model, which accounts for both within-study variability and between-study heterogeneity. Therefore, these effect measures were reasonably integrated and analyzed jointly to ensure the comprehensiveness of the analysis and the maximization of statistical efficacy. Heterogeneity was evaluated through Q test and I² statistic. If P ≥ 0.1 and I² ≤ 50%, a fixed-effect model was used; if P < 0.1 or I² > 50%, a random-effect model was selected. Subgroup analyses were performed based on the number of adjusted covariates (≤2 vs. >2) and adjustment for corticosteroid use (adjusted vs. not adjusted). To assess the robustness of the results, sensitivity analysis was performed using the exclusion method for each article. Publication bias was evaluated by drawing funnel plots and combined through Begg’s test and Egger’s test.

## 3 Results

### 3.1 Study characteristics

**Figure 1:**
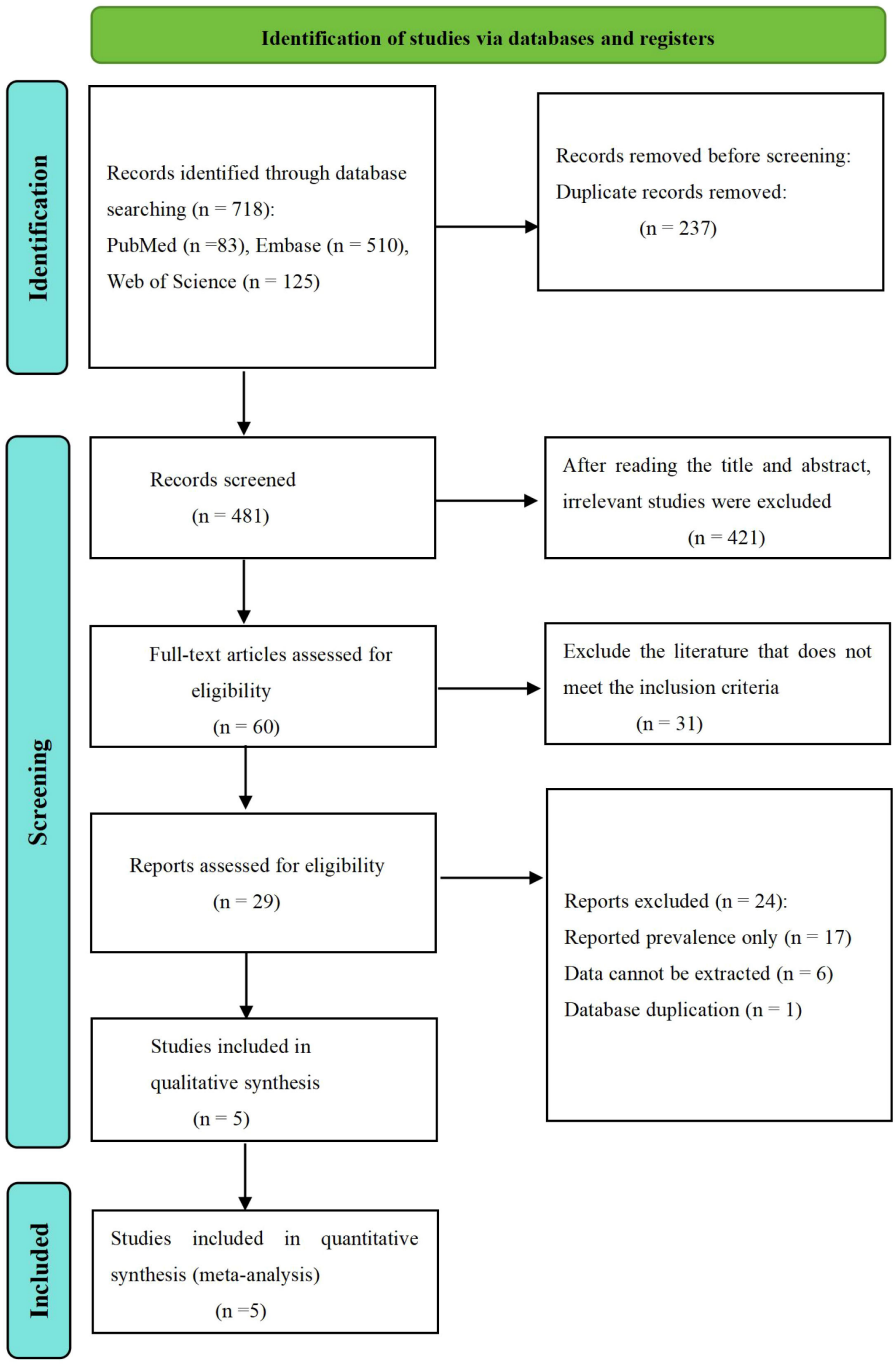
Flowchart of database search and study inclusion.

Through systematic search and citation tracing, a total of 718 relevant records were initially obtained. After removing duplicates, 481 records were retained for the initial screening of titles and abstracts, and 421 studies that did not meet the inclusion criteria were excluded. The remaining 60 documents were re-screened in full text, and after independent assessment and discussion, 55 studies were ultimately excluded, and a total of 5 studies (17–21) that met the criteria were included for meta-analysis (**Figure 1**).

**Table 1** summarizes the basic characteristics of the five studies included in the meta-analysis. These studies were published between 2016 and 2025, and were mainly from South Korea, China and the United States. The study designs were all retrospective cohort or case-control studies. The total sample size was 5516709 and the sample sizes of each study ranged from 554 to 5487712. The study by Chan et al. (20) only included individuals under the age of 18 and did not report the average age. The average age range of the participants in the other studies was 40.3 to 70 years old. The proportion of females ranged from 57.4% to 90.8%. All studies reported the adjusted effect values after multiple variable adjustments, accounting for age and gender. Some studies further included covariates such as body mass index (BMI), smoking, drinking, region, socioeconomic factors, and corticosteroid use. In terms of data sources, except for the study by Becerra et al. (21) which was based on the patients visiting a single clinic, all other studies relied on large-scale epidemiological or medical management databases. According to the ROBINS-E tool, all five included studies had an overall moderate risk of bias (**Table 2**).

**Table 1:** The basic characteristics of the included studies.

References	Study duration	Country	Study design	Sample size	Sex (male/female)	Mean age(years)	SLE definition	Glaucoma definition	Adjustment for covariates
Kim et al., 2025 (18)	From 2008 to 2022	Korea	Retrospective cohort	19364	2920 / 16444 (84.9)	40.08 ± 17.06	ICD-10 M32 + RID V136	ICD-10 H40/H42, ≥2 outpatient visits	Age, Sex, Residence, Income, Comorbidities, CCI, Corticosteroid use, BMI, Smoking, Alcohol
Chan et al., 2016 (20)	From Jan 2003 to Dec 2008	China	Retrospective Cohort	5487712	SLE: 130/774 (85.6)	NR, All < 18	ICD-9-CM 710.0 + ACR 1997	ICD-9-CM 365, ≥3 claims	Age, Sex
Hsu et al., 2020 (19)	From Jan 2000 to Dec 2012	China	Retrospective Cohort	5593	649/5082 (90.8)	40.3	ICD-9-CM 710.0 + ACR 1997	ICD-9-CM 365 (glaucoma); also analyzed POAG (365.11)	Age, Sex, Socioeconomic status, Geographic region
Bai et al., 2023 (17)	From 2000 to 2012	China	Retrospective Cohort	3486	591/2895 (83.0)	40.9	ICD-9-CM 710.0 + EULAR/ACR 2019, ≥3 outpatient or 1 inpatient	ICD-9-CM 365, ≥1 inpatient or 3 outpatient ophthalmic visits	Age, Sex, Hypertension, Hyperlipidemia, Diabetes, COPD, Chronic kidney disease, Chronic liver disease, Corticosteroids use
Becerra et al., 2025 (21)	From Jan 2005 to Dec 2015	USA	Retrospective Case-Control	554	236/318 (57.4)	70	ICD-9 codes	Normal-tension glaucoma, ICD-9 codes + chart review confirmed	Age, sex

BMI, body mass index; CCI, Charlson Comorbidity Index; COPD, chronic obstructive pulmonary disease; NR, Not Reported; OR, Odds Ratio; RR, Risk Ratio; ROR, Reporting Odds Ratio; IRR, Incidence Rate Ratio; HR, Hazard Ratio; ICD, International Classification of Diseases; RID, Rare and Intractable Disease; ACR, American College of Rheumatology; USA, the United States of America; SLE, Systemic Lupus Erythematosus; POAG, Primary Open-Angle Glaucoma; EULAR, European League Against Rheumatism.

**Table 2:** Summary of bias assessment using the ROBINS-E tool.

Study	D1	D2	D3	D4	D5	D6	D7	Overall
Kim et al., 2025 (18)	Low	Low	Low	Low	Low	Moderate	Low	Moderate
Chan et al., 2016 (20)	Moderate	Low	Low	Low	Low	Moderate	Low	Moderate
Hsu et al., 2020 (19)	Low	Low	Low	Low	Low	Moderate	Low	Moderate
Bai et al., 2023 (17)	Moderate	Low	Moderate	Low	Low	Moderate	Low	Moderate
Becerra et al., 2025 (21)	Low	Moderate	Low	Low	Moderate	Low	Low	Moderate

Domains:

D1: Bias due to confounding.

D2: Bias in selection of participants.

D3: Bias in classification of exposures.

D4: Bias due to deviations from intended exposures.

D5: Bias due to missing data.

D6: Bias in measurement of outcomes.

D7: Bias in selection of the reported result.

The color shadings correspond to the risk of bias evaluation results of included studies: light green represents low risk of bias, and yellow represents unclear risk of bias.

### 3.2 Meta-analysis

**Figure 2:**
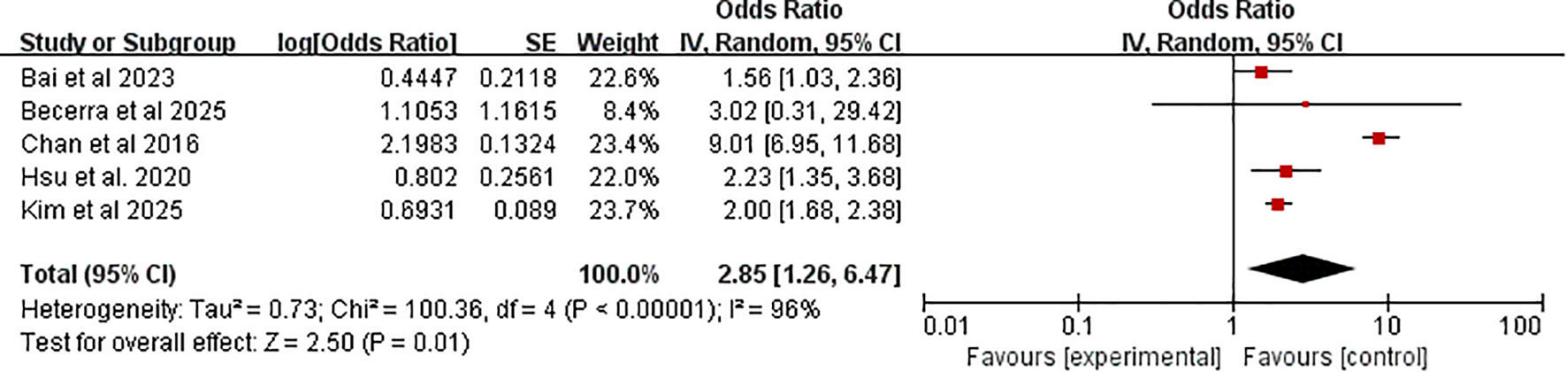
Forest plots of glaucoma risk in Systemic Lupus Erythematosus.

This study ultimately included five studies, involving 5516709 participants. Heterogeneity test results: I² = 96%, *P* < 0.00001, a random effects model was used for the pooled analysis. Meta-analysis showed that the risk of glaucoma in patients with SLE was significantly higher than that in the general population (OR = 2.85, 95% CI: 1.26–6.47, P = 0.01) (**Figure 2**).

The subgroup analysis showed that when the number of adjusted covariates was greater than 2, the analysis results of the association between SLE and the risk of glaucoma indicated that the pooled OR value was 1.95 (95% CI: 1.68–2.28, P < 0.00001), and the difference was statistically significant. When the number of covariates was ≤ 2, the analysis results of the association between SLE and the risk of glaucoma indicated that the pooled OR was 8.88 (95% CI: 6.87–11.50, P < 0.00001), and the difference was statistically significant (**Table 3**).

**Table 3:** The pooled results of each subgroup.

Subgroup analysis	No. of studies	Heterogeneity		OR (95% CI)	P-value
		*I^2^*	*P*		
Number of adjusted covariates					
n >2	3	0%	0.48	1.95 (1.68-2.28)	< 0.00001
n ≤ 2	2	0%	0.35	8.88 (6.87-11.50)	< 0.00001
Corticosteroid adjustment					
Adjusted for corticosteroid use	2	14%	0.28	1.93 (1.64-2.26)	< 0.00001
Unadjusted for corticosteroid use	3	92%	< 0.00001	4.26 (1.28-14.17)	0.02

The second subgroup analysis was performed based on whether corticosteroid use was adjusted. Two studies adjusted for corticosteroid use, while three did not. The pooled OR was 1.93 (95% CI: 1.64–2.26, P < 0.00001) in the adjusted subgroup and 4.26 (95% CI: 1.28–14.17, P = 0.02) in the unadjusted subgroup (**Table 3**).

### 3.3 Sensitivity analysis

**Figure 3:**
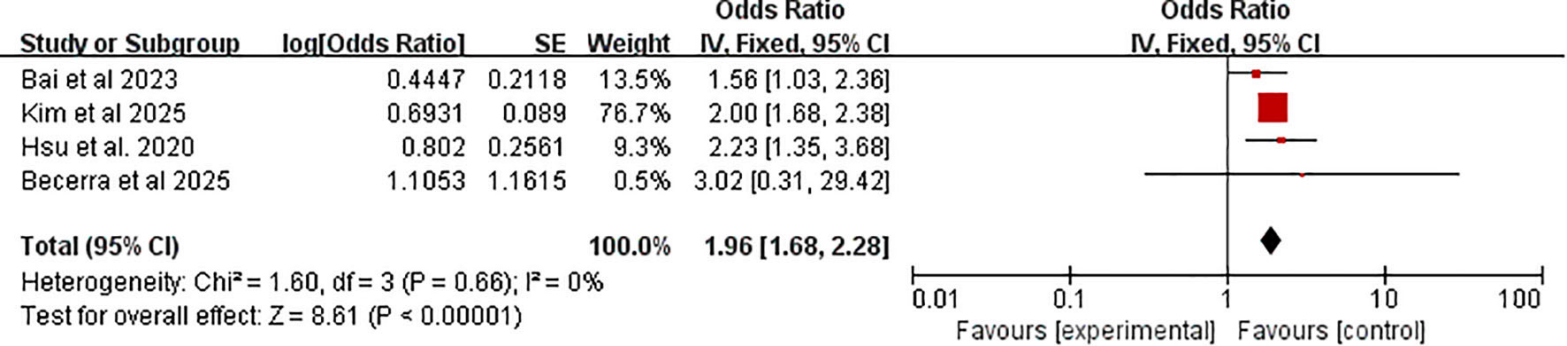
Forest plots of glaucoma risk in Systemic Lupus Erythematosus after excluding the study by 20.

A sensitivity analysis was conducted using the stepwise exclusion method. The results showed that after excluding any single study, the pooled OR and its 95% CI did not undergo significant changes, indicating that the overall association effect obtained in this study has good robustness. After excluding the study by Chan et al. (20), the heterogeneity significantly decreased (I² decreased from 96% to 0%), suggesting that this study might be the main source of high heterogeneity in this Meta-analysis (**Figure 3**).

### 3.4 Publication bias

**Figure 4:**
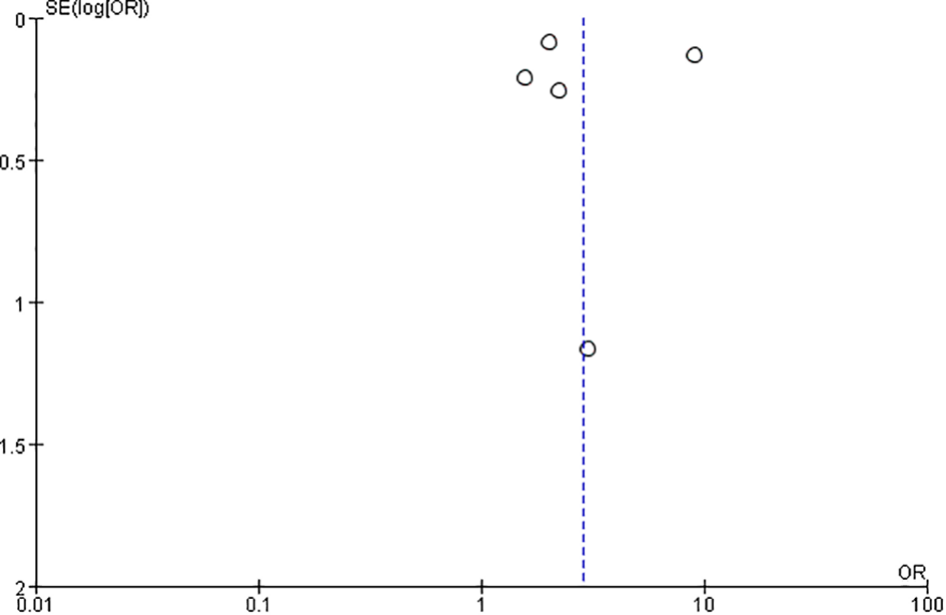
Funnel plots of glaucoma risk in Systemic Lupus Erythematosus.

The funnel plot showed a roughly symmetrical distribution of study points (**Figure 4**). The results of Begg’s test (P = 1.000) and Egger’s test (P = 0.370) did not suggest significant publication bias. However, with only five included studies, these tests have limited statistical power, and the funnel plot should be interpreted with caution.

## 4 Discussion

This study was the first systematic review and meta-analysis conducted on the association between SLE and the risk of glaucoma. By integrating five studies that met the inclusion criteria (with a total sample size of n = 5516709), the relative risk of glaucoma in SLE patients was systematically evaluated. The pooled analysis results showed that the combined risk of glaucoma in SLE patients was significantly higher than that in the general population, and this association was statistically significant. This provides important evidence-based medical evidence for the long-term complication management of SLE patients.

The sensitivity analysis results showed that the study by Chan et al. (20) was the main source of heterogeneity in this Meta-analysis. This finding warrants a careful discussion beyond mere statistical consideration. It adopted standard diagnostic definitions and was nationally representative with high data integrity. However, unlike the other included studies, which predominantly enrolled adult or mixed adult populations, Chan et al. (20) exclusively included pediatric SLE patients (all under 18 years of age). Existing evidence indicated that compared with adult SLE, childhood SLE typically showed higher disease activity, more severe clinical manifestations, and more extensive organ involvement (22). Eye complications (such as glaucoma) may be more prominent in pediatric patients. Long-term glucocorticoid treatment is a common approach in the management of pediatric SLE, and glucocorticoids are recognized as a risk factor for secondary glaucoma (23). Considering that the anterior chamber structure of children has not fully developed, they are significantly more sensitive to the increased resistance to aqueous humor outflow caused by glucocorticoids compared to adults. Moreover, due to the more severe condition of children with SLE, the intensity of glucocorticoid treatment for them is usually higher (24, 25). These factors collectively explain the substantially higher effect size observed in the Chan et al. (20) study. Rather than treating this heterogeneity as a methodological flaw, we interpret it as an important clinical phenomenon: the association between SLE and glaucoma may be considerably stronger in pediatric populations. This finding highlights the urgent need for age-stratified analyses and dedicated ophthalmic surveillance protocols for children with SLE. To prevent irreversible optic nerve damage and visual field defects, clinicians should prioritize systematic ophthalmic follow-up for children with SLE, and should particularly conduct regular intraocular pressure monitoring to enable early identification of glaucoma signs and timely implementation of intervention measures.

The subgroup analysis indicated that the association between SLE and glaucoma was statistically significant after adjusting for different numbers of covariates. However, the effect size reported in studies with fewer adjustments (≤2) was significantly higher than that in studies with more adjustments (>2). This suggests that there is a clear confounding effect in observational studies. If such confounding factors are not adequately corrected, the strength of the association between SLE and glaucoma will be overestimated. Glucocorticoids, as the basic treatment for SLE and a clear risk factor for glaucoma, may be the core confounding variable in this association.

The subgroup analysis based on corticosteroid adjustment further supported this notion. Studies that adjusted for corticosteroid use yielded a lower estimate (OR = 1.93), whereas those that did not adjust for corticosteroid use showed a substantially higher estimate (OR = 4.26). This striking difference suggests that corticosteroid use is a major confounder in the association between SLE and glaucoma. Failure to account for corticosteroid exposure may lead to a significant overestimation of the true effect size. Therefore, future studies should routinely collect and adjust for detailed corticosteroid use information (including cumulative dose, duration, and route of administration) to more accurately estimate the independent effect of SLE on glaucoma risk. Additionally, differences in the disease activity of SLE may also affect the risk of developing glaucoma. Therefore, future studies need to systematically adjust these key confounding variables to more accurately assess the independent impact of SLE on glaucoma.

The large-scale retrospective cohort study conducted by Kim et al. (18) in South Korea demonstrated that the incidence of glaucoma in the SLE patient group was significantly higher than that in the control group (11.34% vs 3.74%, p < 0.0001), and this result was consistent with the conclusion of our study. Additionally, the study found that younger (under 40 years old) and female patients had a higher risk of glaucoma, and long-term use of corticosteroids would further increase this risk. The research by Bai et al. (17) also confirmed that the risk of glaucoma in SLE patients was significantly increased, with a risk ratio approximately 1.56 times that of the control group. This was more prominent in male patients. Most studies suggest that the reason for the increased risk of glaucoma in SLE patients is the use of glucocorticoids. However, in the above two studies, even after adjusting for demographic characteristics, comorbidities, and medication factors, the risk of SLE-related glaucoma remained significant. This suggests that the unique immunopathological process of SLE may independently contribute to the pathogenesis of glaucoma.

The mechanism of glaucoma in patients with SLE is diverse and is closely related to the pathological activities of the disease itself and the therapeutic drugs. It can mainly be classified into types such as steroid-induced glaucoma, open-angle glaucoma, secondary angle-closure glaucoma, and neovascular glaucoma. The mechanism of its occurrence may be intertwined with multiple factors including immunity, blood vessels, inflammation, and pharmacology. Glaucoma induced by glucocorticoids is the most common iatrogenic mechanism. Long-term use of glucocorticoids can activate the glucocorticoid receptors in trabecular meshwork cells, thereby increasing the synthesis of extracellular matrix (such as glycosaminoglycans and fibronectin) and reducing their degradation, ultimately leading to narrowing of the trabecular meshwork space and increased resistance to aqueous humor outflow (26, 27). At the same time, glucocorticoids upregulate the expression of the MYOC gene, causing abnormal deposition of myosin fibers, which further hinders the drainage of aqueous humor (26, 28). If the medication is used for an excessively long period (for example, more than 17 months), the aforementioned damage may be irreversible (29). It is worth noting that the combined use of calcineurin inhibitors and other immunosuppressants may exert a certain protective effect on the increase in intraocular pressure caused by glucocorticoids by interfering with the relevant signaling pathways (9). Therefore, in the long-term hormone treatment of SLE, it is essential to pay attention to intraocular pressure monitoring and consider adopting hormone protection strategies such as calcineurin inhibitors. Open-angle glaucoma is the most common type of glaucoma, and its occurrence and development in SLE patients involves multiple pathological and physiological mechanisms (30). Chronic inflammation and vascular dysfunction can damage the microcirculation of the retina and optic nerve head, affecting the blood supply and metabolism of retinal ganglion cells (31). The core molecular mechanisms include axonal transport disorders, activation of apoptotic pathways, oxidative stress, and excitotoxicity, which are all neurodegenerative processes. At the same time, activated glial cells further exacerbate neuroinflammation and synaptic loss by releasing pro-inflammatory factors such as TNF-α (32). The autoimmune reactions associated with SLE may also directly or indirectly contribute to the damage of retinal ganglion cells (30). Therefore, this process is essentially a multi-pathway neurodegenerative pattern involving inflammation, abnormal blood vessels, glial cell responses, and autoimmunity. Secondary angle-closure glaucoma results from choroidal lesions and ciliary body edema. During the active phase of SLE, factors such as immune complex deposition, vascular damage, and hypoproteinemia can cause a significant increase in choroidal vascular permeability (33). The excessive leakage of fluid into the choroidal space can lead to ciliary-body-choroidal exudative edema, which, by pushing forward, causes the lens-iris septum to move forward and results in mechanical closure of the anterior chamber angle. Eventually, this leads to an acute increase in intraocular pressure (34). Neovascular glaucoma is caused by the vascular proliferation driven by retinal ischemia. Retinal vascular lesions (such as vasculitis or thrombosis) induced by SLE can lead to retinal ischemia, which in turn stimulates the massive release of vascular endothelial growth factor (VEGF) (35). VEGF causes abnormal new blood vessel membranes to form in the iris and the angle of the eye. These blood vessels eventually block the drainage channels of the aqueous humor, leading to a sharp increase in intraocular pressure and the development of refractory neovascular glaucoma (36). In conclusion, the occurrence of glaucoma in patients with SLE is not caused by a single factor, but rather is a pathological process involving multiple mechanisms. The immune-inflammatory activity of the disease itself can affect intraocular pressure through causing ischemia of the ciliary retina, chronic damage to the neurovascular unit, and choroidal edema. At the same time, the key therapeutic drug, glucocorticoid, directly increases the resistance to aqueous humor outflow by altering the structure and function of the trabecular meshwork. Therefore, the ophthalmic management of patients with SLE should adopt a comprehensive approach: while actively controlling the activity of the underlying disease and carefully adjusting the dosage of glucocorticoids, regular and comprehensive ophthalmic monitoring (including intraocular pressure, anterior chamber angle, and fundus examination) must be conducted to early identify various risks of glaucoma and promptly take targeted interventions, thereby maximizing the protection of visual function.

This study has certain limitations. Firstly, although we conducted a sensitivity analysis to identify the sources of heterogeneity and the sensitivity analysis results showed that the meta-analysis results were stable, there might still be other sources of heterogeneity due to the differences in sample size, population characteristics (such as race, age, and disease activity), and the adjusted confounding factors among the included studies. Secondly, although we identified the inclusion of pediatric SLE patients as a major source of heterogeneity, the limited number of studies precluded a formal subgroup analysis by age group. Consequently, the pooled estimate derived from this meta-analysis should be interpreted with caution when applied to specific age populations. Thirdly, due to the limited number of included studies, only subgroup analysis was conducted on the number of confounding factors, and other factors were not analyzed. This may lead to certain biases in the meta-analysis results. Finally, all the included studies were of observational design (cohort studies), and residual confounding or reverse causality could not be completely excluded. Additionally, because most included studies did not distinguish between glaucoma subtypes (e.g., open-angle vs. steroid-induced vs. angle-closure glaucoma), our pooled estimate reflects overall glaucoma risk rather than subtype-specific associations.

## 5 Conclusions

In conclusion, the available evidence suggests that patients with SLE may have an increased risk of glaucoma. However, this finding should be interpreted cautiously given the small number of included studies, substantial heterogeneity, and potential residual confounding. It is recommended that future studies be designed rigorously, be prospective, be multi-center, and involve a large sample size. Additionally, detailed collection of SLE disease activity indicators, medication history, and ophthalmic examination data should be conducted to more accurately reveal the underlying mechanisms of the association between the two and provide evidence-based guidance for the monitoring and management of ocular complications in SLE patients.

## Data Availability

The original contributions presented in the study are included in the article/**Supplementary Material**, Further inquiries can be directed to the corresponding author/s.

## Supplementary material

Supplementary Table 1 Search strategies.
