# Supplementary material for: The risk association between systemic lupus erythematosus and glaucoma: a systematic review and meta-analysis
Source: Front Immunol. 2026 Jun 22;17:1808105. doi: 10.3389/fimmu.2026.1808105 (PMC13333522; doi:10.3389/fimmu.2026.1808105)
Supplement: Supplementary Table 1 — Search strategies. [file Table1.pdf]

Table S1 Search strategies

Number	Search words
#1	Lupus Erythematosus, Systemic [MeSH Terms]
#2	Lupus Erythematosus Disseminatus [Title/Abstract]
#3	Systemic Lupus Erythematosus [Title/Abstract]
#4	Libman-Sacks Disease [Title/Abstract]
#5	Disease, Libman-Sacks [Title/Abstract]
#6	Libman Sacks Disease [Title/Abstract]
#7	OR/1-6
#8	glaucoma [MeSH Terms]
#9	glaucomas [Title/Abstract]
#10	OR/8-9
#11	#7 AND #10

1
